# Virulence Factors and Antifungal Susceptibility Profile of *C. tropicalis* Isolated from Various Clinical Specimens in Alexandria, Egypt

**DOI:** 10.3390/jof7050351

**Published:** 2021-04-29

**Authors:** Mohammed A. El-Kholy, Ghada F. Helaly, Ebtisam F. El Ghazzawi, Gamal El-Sawaf, Sherine M. Shawky

**Affiliations:** 1 Department of Microbiology and Biotechnology, Clinical and Biological Sciences Division, College of Pharmacy, Arab Academy for Science, Technology and Maritime Transport (AASTMT), P.O. Box 1029, Alexandria, Egypt; 2 Department of Microbiology, Medical Research Institute, Alexandria University, P.O. Box 1029, Alexandria, Egypt; ghadafahmy@hotmail.com (G.F.H.); ebtelu2@hotmail.com (E.F.E.G.); gamalsawaf@yahoo.com (G.E.-S.); sherineshawky@hotmail.com (S.M.S.)

**Keywords:** candidiasis, antifungal susceptibility, virulence factors, *C. tropicalis*, non-*albicans* *Candida*

## Abstract

Background: The incidence of candidiasis caused by non-*albicans Candida* (NAC) species is increasing. *Candida tropicalis* has emerged as one of the most important NAC species. This study aims to examine the antifungal susceptibility profile and some virulence factors of *C. tropicalis* isolated from various clinical specimens. Methods: A total of 71 *C. tropicalis* isolates from various clinical specimens (69.01%, 18.31%, 9.86%, and 2.82% of isolates were collected from urine, respiratory samples, blood, and skin and soft tissue infections, respectively) from ICU patients in Alexandria, Egypt. The isolates were identified at species level by CHROMagar *Candida* and VITEK 2 compact system. Furthermore, the antifungal susceptibility was determined using the VITEK 2 system AST-YS07 card containing different antifungals. Hemolysin, phospholipase, and proteinase activity and biofilm formation were also tested as virulence factors. Results: Only 30 isolates (42.25%) were non-susceptible (MIC ≥ 4 µg/mL) to fluconazole, of which 28 isolates showed non-susceptibility (MIC ≥ 0.25 µg/mL) to voriconazole. All isolates showed both hemolysin and proteinase activities, while only 9 isolates (12.68%) showed phospholipase production and 70 isolates (98.59%) demonstrated biofilm formation. Strong biofilm production was observed among the blood culture isolates (85.71%), followed by the respiratory and urinary isolates (61.54% and 46.94%, respectively). Conclusions: This study sought to provide useful data on the antifungal susceptibility of *C. tropicalis* isolates from ICU patients suffering from invasive infections with an increased trend towards elevated MICs levels of both fluconazole and voriconazole. Due to the high incidence of systemic candidiasis and antifungal resistance, *C. tropicalis* is emerging as a serious root of infections. Therefore, early and accurate identification of *Candida* species along with susceptibility testing is of utmost importance.

## 1. Introduction

The incidence of mycotic infections has gradually increased over the past few years. *Candida *spp. are among the most common fungal pathogens [1]. *Candida* spp., considered as a part of the normal flora, may cause diseases ranging from superficial candidiasis to life-threatening disseminated infections under several circumstances that compromise host immunity [2,3,4,5]. Although* Candida albicans* is the predominant species involved in both superficial and disseminated infections, there has been a significant increase in the number of infections caused by non-*albicans Candida* (NAC) species [6].

*Candida tropicalis*, *Candida parapsilosis, Candida glabrata*, and *Candida krusei* are the NAC species increasingly reported as both colonizers and pathogens causing nosocomial candidemia. They may account for almost half of all non-superficial *Candida* infections [6,7,8,9]. Among the NAC spp., *C. tropicalis* is frequently isolated from different clinical types of candidiasis [10,11]. It is often related to higher mortality compared to other NAC species and *C. albicans*, principally in neutropenic and cancer patients [12,13].

*C. tropicalis* accounts for a significant proportion of *Candida* bloodstream isolates in tropical regions and in infections of cancer patients. [14]. *C. tropicalis* colonizes various anatomically distinct sites, including the skin and the respiratory, gastrointestinal, and genitourinary tracts. It can also be recovered from the environment, predominantly from surfaces in medical settings [12,15].

*C. tropicalis* has many virulence features that assist in its invasion of host tissues. They include adherence to host tissues as well as medical devices, formation of pseudohyphae to aid in evasion of the host immune defenses, biofilm production, and production of tissue-damaging hydrolytic extracellular enzymes (e.g., proteinases, phospholipases, and hemolysin) [14].

In clinical practice, azoles, fluoropyrimidine, echinocandins, and polyenes are used for treating fungal infections [16]. Azoles are the most frequently used antifungals. They are also studied widely for their pharmacological properties, mode of action, and resistance by microorganisms. Different levels of resistance towards these antifungals are observed in a variety of NAC species [10]. Therefore, greater incidence of systemic candidiasis and resistance to antifungals have become a matter of concern worldwide.

In the present study, we aimed to examine some virulence factors of *C. tropicalis* isolated from various clinical specimens that were collected from several ICU patients admitted to different medical facilities in Alexandria, Egypt, as well as to determine their antifungal susceptibility profiles, with special emphasis on azole resistance.

## 2. Materials and Methods

### 2.1. Sampling

A total of 71 non-duplicate *C. tropicalis* isolates from various clinical specimens were included in this study. Samples were collected from several ICU patients admitted to different medical facilities in Alexandria, Egypt, including the Medical Research Institute (MRI), the Alexandria Main University Hospital, and the Mabaret El Asafra Hospital.

Medical Research Institute, Alexandria University, serial number E/C. S/N. T81/2014, approved on 29/11/2014.

### 2.2. Identification

Identification of all clinical isolates was performed to the species level by culturing on Sabouraud dextrose agar (SDA) at 35–37 °C until growth appeared, followed by Gram staining. The growth from SDA was plated onto BBL™ CHROMagar Candida (BD Diagnostic systems, Heidelberg, Germany). *C. tropicalis* appeared as blue-greenish to metallic-blue colonies on the chromogenic agar. All the strains were subjected to further identification by the VITEK 2 compact system according to the manufacturer’s instructions [17].

### 2.3. Antifungal Susceptibility Testing

Based on the manufacturer’s instructions, antifungal susceptibilities of all isolates were determined using the VITEK 2 compact system (AST-YS07 card; bioMérieux, Marcy l’Etoile, France). Quality control was performed by testing *C. krusei* ATCC 6258 and *C. parapsilosis* ATCC 22019. Furthermore, the VITEK 2 cards containing serial two-fold dilutions of amphotericin B, caspofungin, fluconazole, flucytosine, micafungin, and voriconazole were provided by the manufacturer [17]. The interpretation of the *C. tropicalis* antifungal susceptibility testing results was based on the CLSI species-specific clinical breakpoints (SS-CBPs) (CLSI M27-S4) for fluconazole, voriconazole, caspofungin, and micafungin [18], and on the epidemiological cut-off values (ECVs) for flucytosine and amphotericin B [19].

### 2.4. Assessment of Virulence Factors

Hemolytic activity. *C. tropicalis* isolates were screened for hemolysin production by a previously described method [20]. Additionally, a standard inoculum containing approximately 10^8^ *Candida* cells/mL was prepared from the tested strains and a control strain (*C. albicans* ATCC 90028). A volume of 10 μL of this standard inoculum was deposited onto SDA supplemented with glucose (3%) and fresh sheep blood (7%). The plates were then incubated at 37 °C in 5% CO_2_ for 48 h. When viewed in transmitted light, positive hemolytic activity was indicated by the presence of a distinct translucent halo around the inoculum site. The hemolytic activity (Hz) was measured using the method described by Price et al., 1982, where the ratio of the diameter (in mm) of the colony to that of the translucent zone of hemolysis was calculated [21]. All assays were carried out in triplicate in addition to classifying the hemolytic activity into four categories based on the obtained Hz score. No enzymatic activity was detected if the Hz was 1.0, Hz between 0.999 and 0.700 meant low enzymatic activity, whereas moderate activity was indicated by Hz between 0.699 and 0.400, and low Hz values between 0.399 and 0.100 corresponded to high hemolytic activity [20].

Proteinase production. Production of extracellular proteinase was assessed by means of a previously described method [22]. Yeast suspensions of 10 μL (1.0 × 10^6^ CFU/mL) were spot-inoculated on bovine serum albumin (BSA) agar plates (20 mL of a solution containing 0.04 g Mg_2_SO_4_·7H_2_0, 0.5 g K_2_HPO_4_, 1 g NaCl, 0.2 g dried extract of yeast, 4 g glucose, and 0.05 g BSA mixed with 180 mL of molten agar) and incubated at 37 °C for 5 days. Then, the plates were fixed with 20% trichloroacetic acid, stained with 1.25% amido black dye, and destained with 12.5% acetic acid. The clear zone was measured, which corresponds to the hydrolysis of the BSA present in the medium. Proteinase activity (Prz value) was calculated in terms of the ratio of the diameter of the colony to the total diameter of the colony plus the zone of solubilization.

A Prz value of 1 indicated no proteinase production; Prz < 1 indicated proteinase activity. That is, the lower the Prz value, the higher the proteinase activity. This procedure was repeated at least three times, and all assays were carried out in triplicate. Accordingly, the proteinase activity was scored into four categories: a Prz of 1.0 indicated no enzymatic activity, a Prz between 0.999 and 0.700 indicated low enzymatic activity, Prz between 0.699 and 0.400 corresponded to moderate activity, and low Prz values between 0.399 and 0.100 meant high proteinase activity [22].

Phospholipase production. The phospholipase activity of *C. tropicalis* was also detected by previously described methods [21,23] with minor modifications. Yeast suspensions of 1.0–2.0 × 10^6^ yeasts/mL were transferred to the test plates (SDA supplemented with 1 M sodium chloride, 0.005 M calcium chloride, and 8% sterile egg yolk (Oxoid Ltd., Basingstoke, UK)). The plates were then incubated at 37 °C for 48 h in a humid chamber. Further incubation was carried out up to 5 days as more isolates demonstrated phospholipase activity upon prolonged incubation. The zone and colony sizes were calculated each day.

A precipitation zone was produced around the colony due to hydrolysis of lipid substrates present in the egg yolk. Diameters of the colonies and precipitation zones were measured, and phospholipase activity (Pz value) was determined by the ratio of the diameter of the colony to the total diameter of the colony plus the precipitation zone [21]. This procedure was repeated at least three times, and all assays were carried out in triplicate. Based on the scores, the phospholipase activity fell into four categories: a Pz of 1.0 indicated no enzymatic activity, a Pz between 0.999 and 0.700 indicated low enzymatic activity, Pz between 0.699 and 0.400 corresponded to moderate activity, and low Pz values between 0.399 and 0.100 meant high phospholipase activity [24].

### 2.5. Determination of Biofilm Formation

Visual tube method (qualitative method): Assessment of biofilm formation by *C. tropicalis* was performed as previously described [25]. A volume of 5 mL Sabouraud dextrose broth (SDB), supplemented with 8% glucose, in screw-capped conical polystyrene tubes was inoculated with an overnight growth of *C. tropicalis* and incubated at 35 °C for 48 h without agitation. The tubes were then decanted, washed twice with distilled water, and dried. The dried tubes were stained with 1% crystal violet, and the excess stain was washed with distilled water. A development of a visible film, lining the walls and bottoms of the tubes, was considered as biofilm formation. Biofilm production was scored as negative (−), weak (+), moderate (++), or strong (+++). *Staphylococcus epidermidis* ATCC 35984 and *C. albicans* ATCC 90028 were used as positive and negative controls, respectively.

Spectrophotometric microplate method (semiquantitative method): In the beginning, 200 μL of a standardized cell suspension (10^7^ cells/mL in SDB) was transferred into each well of a microtiter plate with a pipette. After 48 h of incubation, the total biofilm biomass was quantified via crystal violet staining [26]. The medium was fully aspirated, and the non-adherent cells were removed by washing the biofilms once with 200 μL of PBS. Then, the biofilm was fixed with 200 μL of methanol (100% *v*/*v*), which was removed after 15 min of contact. Subsequently, the microplates were left to dry at room temperature, and 200 μL of crystal violet (0.1% *v*/*v*) was added to each well and incubated for additional 5 min. Afterwards, the wells were gently washed twice with 200 μL of sterile distilled water, and 200 μL of acetic acid (33% *v*/*v*) was added to release and dissolve the absorbed stain. Absorbance of the obtained solution was read in triplicate in a microplate reader at 620 nm (Tecan Infinite F50 Microplate Reader; Tecan Group Ltd., Mannedorf, Switzerland).

On the basis of the cut-off optical density (ODc), the biofilm formations of different strains were classified into groups. For test microplates, the ODc was defined as three standard deviations above the mean OD of the negative control. Isolates having an OD lower than the cut-off, less than 2 times the cut-off, and 2–4 times the cut-off were considered as non-adhered (non-biofilm forming), weakly adhered, moderately adhered, and strongly adhered, respectively [27]. ODc: = 0.06, 2× = 0.12, 4× = 0.24.

## 3. Results

In the present study, a total of 71 non-duplicate *C. tropicalis* isolates were collected from urine (49, 69.01%), respiratory samples (13, 18.31%), blood (7, 9.86%), and skin and soft tissue infections (2, 2.82%). Thirty isolates (42.25%) were non-susceptible (resistant or dose-dependent susceptibility, SDD) to fluconazole (MIC ≥ 4 µg/mL). Out of these 30 isolates, 28 were non-susceptible (resistant or SDD) to voriconazole (MIC ≥ 0.25 µg/mL) as well, while only 2 isolates were sensitive to voriconazole. Moreover, all isolates were susceptible to amphotericin B, flucytosine, and echinocandins (caspofungin and micafungin) (Table 1).

According to CLSI (M27-S4) fluconazole breakpoints (S ≤ 2, SDD = 4, R ≥ 8 µg/mL), 57.75% of the isolates were sensitive to fluconazole with MIC ≤ 1 µg/mL, and 5.63% displayed SDD to fluconazole with MIC 4 µg/mL, while 36.62% were resistant to fluconazole with MIC ≥ 8 µg/mL (Table 2). As for the voriconazole breakpoints (S ≤ 0.125, SDD = 0.25–0.5, R ≥ 1 µg/mL), 60.56% of the isolates were voriconazole sensitive with MIC ≤ 0.125 µg/mL, and 32.4% showed SDD to voriconazole with MIC 0.25–0.5 µg/mL, whereas 7.04% were voriconazole resistant, with MIC ≥ 1 µg/mL (Table 2). Cross-resistance to azoles (fluconazole and voriconazole) was found in 7.04% of isolates.

**Figure 1:**
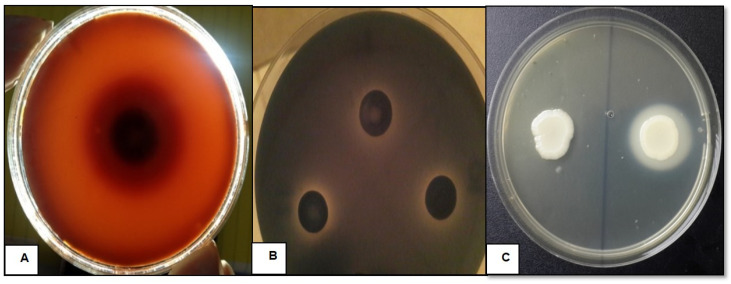
**(A**) *C. tropicalis* on blood SDA showing dual hemolytic zones; (**B**) proteinase activity of *C. tropicalis* isolates; (**C**) *C. tropicalis* isolates showing positive (right) and negative (left) phospholipase activities on egg yolk-enriched SDA.

All *C. tropicalis* isolates showed positive hemolytic activities demonstrated by hemolysis on human blood SDA. Almost all isolates had a moderate hemolytic activity (Hz) in the range of 0.958–0.400 with a mean of 0.532 ± 0.068, whereas two isolates demonstrated low activities. After 48 h of incubation, the hemolytic activity was higher than after 24 h. At 24 h post inoculation, only alpha hemolysis was observed surrounding the inoculum sites of all strains. However, after further incubation up to 48 h, the zone of hemolysis was enlarged, showing dual zones of alpha and beta hemolysis (Figure 1A).

All *C. tropicalis* isolates showed a positive proteinase activity, producing translucent zones around the colonies on BSA agar plates (Figure 1 B). Proteinase activity (Prz) was characterized as low, ranging between 0.933 and 0.600 with a mean of 0.804 ± 0.061. Only four isolates, which were isolated from urine samples, showed moderate activities.

However, phospholipase activity was demonstrated in 9/71 (12.68%) of the tested *C. tropicalis* isolates (Figure 1C). Positive phospholipase activity (Pz value) was characterized as low, ranging between 0.9 and 0.6 with a mean of 0.816 ± 0.092. Only one sample, isolated from the respiratory tract, showed moderate activity. We noticed that the phospholipase activity increased during prolonged incubation, resulting in opaque precipitation zones around the colonies.

**Figure 2:**
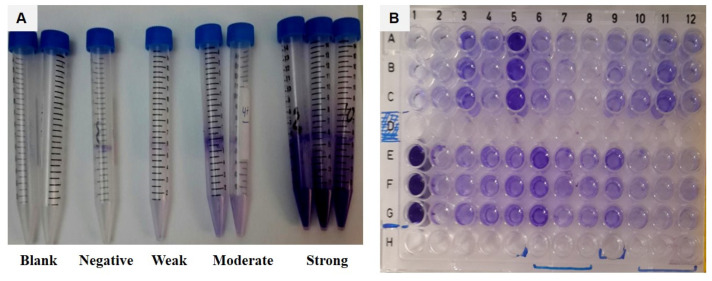
Determination of biofilm production by (**A**) visual method and (**B**) spectrophotometric method.

The study of biofilm production revealed no major discrepancies between the results obtained by the visual tube and spectrophotometric microplate methods (Figure 2). All tested isolates showed biofilm production, except one. The isolates were classified as strong, moderate, and weak biofilm producers with respect to the sample type (Table 3). Nonetheless, no relationship could be detected between biofilm production and azole resistance (Table 4).

## 4. Discussion

In patients with *Candida* infections, *C. tropicalis* falls among the most frequently detected and isolated species. It is associated with hematological malignancy and urinary tract infections [12]. Previous studies showed that *C. tropicalis* was the most common NAC species amongst *Candida* bloodstream isolates [6,28]. Candiduria significantly increases the risk of death in low birth weight infants [29]. Data concerning the profile and antifungal susceptibility of *Candida* spp. are relatively few in Arab countries and other countries in the region.

The current study aimed to expand the knowledge concerning NAC species in Egypt, focusing on *C. tropicalis* as it represents one of the most common NAC species. The current study included 71 *C. tropicalis* isolates from various clinical specimens from different ICUs in Alexandria, Egypt. These isolates were identified to species level using Chromogenic media and VITEK 2 compact system, which demonstrated similar results, denoting that they represent valuable methods for identification of non-*albicans Candida* species. Concerning the rate of azole susceptibility, 5.63% and 32.39% of the isolates were non-susceptible, and 36.62% and 7.04% were resistant to fluconazole and voriconazole, respectively, where the latter isolates were also found cross-resistant to both agents. However, no resistance to echinocandins, amphotericin B, nor 5-flucytosine was detected.

In Egypt, few studies discussed the prevalence and antifungal susceptibility of *C. tropicalis*. Two studies reported no resistance against fluconazole in Egypt [30,31]. In another study, involving 16 *C. tropicalis* isolates, 25% of the isolates were resistant to fluconazole, 12% were resistant to voriconazole, and one isolate (6%) was resistant to amphotericin B [32]. However, it is worth mentioning that this study applied the CLSI document M44-A (2004). In a study conducted by Shawky et al., 2017, on a total of 1023 *Candida* isolates, it was indicated that *C. tropicalis* was the second most frequently isolated NAC species, as the azole resistance percentages were 24.6% and 17.5% to fluconazole and voriconazole, respectively [33].

Different Arab countries reported discrepant results regarding azole susceptibility. A low incidence of azole resistance was reported formerly among *C. tropicalis* strains isolated from Tunisian hospitals [34]. On the contrary, a Saudi study reported a high rate of fluconazole and voriconazole resistance (62.5% and 25%, respectively) among *C. tropicalis* isolates [35].

Based on a study conducted in China, it was reported that 11.6% and 9.5% of their *C. tropicalis* isolates were non-susceptible to fluconazole and voriconazole, respectively, which is much lower than the results in the present study for voriconazole. While 7.1% of the isolates showed cross-resistance to both azoles, the study also showed that 98.9% of the isolates were sensitive to 5-flucytosine, and all isolates were sensitive to caspofungin, micafungin, and amphotericin B [36]. Similar results were also reported in a study from Taiwan [37].

Contradictions regarding azole susceptibility rates might be due to the difference in sample sizes, methods used for determination of antifungal susceptibility, and breakpoints used for interpretations, in addition to different uses of azoles in prophylaxis therapy among countries or institutions [38].

Virulence factors, such as hemolytic activity, biofilm formation, and production of extracellular proteinases and phospholipases, may be involved in the pathogenic process of *C. tropicalis* [39]. In the study at hand, all *C. tropicalis* isolates showed hemolytic activity, and the majority of tested isolates showed moderate hemolytic activity. Many previous studies had also shown similar results. In a previous study carried out by Yu et al., 2015, the majority of their *C. tropicalis* isolates showed positive hemolytic activities at each time point (24, 48, and 72 h), with the highest one at 72 h [40]. Another previous study reported that 93.33% of the tested *C. tropicalis* isolates showed hemolytic activities, of which 74% exhibited a moderate hemolytic index [41]. On the contrary, a lower hemolytic activity in *C. tropicalis* was also reported previously [42].

Many NAC species obtained from clinical sources exhibit varying ability to produce up to two different types of hemolysin, alpha and beta, causing incomplete and complete hemolysis of the blood agar medium, respectively [43]. The results of this study showed that all the isolates demonstrated both alpha and beta hemolysis. Dual zones of hemolysis were also observed and reported in other studies [44,45]. Alpha- and beta-hemolytic activities may be the result of two or more different hemolytic factors sequentially produced by the yeasts.

All the tested isolates in this study showed low proteinase activity, except four strains, isolated from urine samples, which showed moderate activity. Our results are in accordance with previously published studies [46,47,48]. On the contrary, former studies revealed that 40–87% of *C. tropicalis* strains were able to yield proteinase activity [24,49,50]. While one study reported that the majority (60%) of isolates did not show proteolytic activity when assessed by a semi-quantitative method (the plate method), the proteolytic activity profile attained for the semi-quantitative method was different from the quantitative method. The latter method showed activity of all *C. tropicalis* isolates. Furthermore, the protease activity of *C. tropicalis* was better detected by the quantitative assay [51]. When comparing aspartyl proteinase activities at different time points in the present study, it was found that the activity was higher after 72 h incubation than after 24 or 48 h. Our finding is in agreement with a previous report [40].

Phospholipase production is considered as one of the virulence factors of *C. tropicalis*. We detected phospholipase enzymatic activity in 12.68% of the tested isolates. Nearly similar results were reported in previous studies; Negri et al. [50] and Udayalaxmi et al. [52] reported that 14.29% and 15.8% of their *C. tropicalis* strains were phospholipase producers, respectively.

Lower percentages of isolates showing phospholipase enzymatic activity were reported previously [48,53]. High phospholipase activity in *C. tropicalis* [49,54] as well as no activity at all [40] were also reported in previous studies. The inconsistencies observed regarding *C. tropicalis* spp. phospholipase activity could be attributed to several factors, such as method of media preparation, difference in incubation temperature and duration. Discrepancies could also be attributed to the plate method used as it may not detect the activity in low-phospholipase-producing strains. It is postulated that more sensitive methods are needed to detect lower amounts of phospholipases produced by NAC species [55].

Biofilm production represents the most important virulence factor of NAC species [56]. *C. tropicalis* strains are able to form biofilms on silicone, which has an important clinical impact. Additionally, biofilm-associated infections are difficult to treat, representing a source of reinfections [50]. The mortality rates in patients infected by biofilm-forming isolates are greater than those infected by non-biofilm-forming isolates [57].

In the present study, almost all tested isolates (98.6%) showed biofilm production. Overall, 53.52%, 35.21%, and 9.86% were strong, moderate, and weak biofilm producers, respectively.

Similar results were observed in a study where more than 80% of *C. tropicalis* isolates were strong biofilm producers, and only 7% were low biofilm producers [58]. Guembe et al., 2017, reported parallel findings [59]. On the contrary, a lower percentage of *C. tropicalis* biofilm producers has also been reported [49]. Variations among *C. tropicalis* strains concerning biofilm formation could be due to physiological differences between strains according to the origin of the isolates [60]. No relationship was detected between biofilm production and azole resistance in the present study, which is in agreement with Furlaneto-Maia et al., 2008 [45]. Nevertheless, another study contradicts this observation and reported a correlation between biofilm production and resistance to 5-flucytosine and fluconazole [61].

In the present study, the number of strong biofilm producing isolates was higher among blood culture isolates, followed by respiratory and urinary tract isolates. This finding agrees with previous studies, where *C. tropicalis* isolated from blood and urine demonstrated high biofilm production capacity [59,62].

According to these results, the expression of virulence factors can vary depending on the *Candida* strain as well as the site of isolation.

In conclusion, this study has attempted to provide useful data on the antifungal susceptibility of *C. tropicalis* isolates from ICUs patients in Alexandria, Egypt. It has also indicated the importance of performing antifungal susceptibility tests, along with an increased trend towards elevated MIC levels of both fluconazole and voriconazole. This study has also demonstrated different virulence factors. More locally relevant epidemiological studies should be carried out to determine the changing epidemiology of candidiasis, highlighting the need for close monitoring of the distribution and susceptibility profile in order to optimize therapy and outcome.

## Figures and Tables

**Table 1:** Antifungal susceptibility profile of *C. tropicalis* clinical isolates.

Antifungal Agent	Sensitivity	Dose-Dependent Susceptibility	Resistance
	*n* (%)	*n* (%)	*n* (%)
*(I) Azoles*			
Fluconazole	41(57.75)	4(5.63)	26(36.62)
Voriconazole	43(60.56)	23(32.39)	5(7.04)
*(II) Echinocandins*			
Caspofungin	71(100)	0(0)	0(0)
Micafungin	71(100)	0(0)	0(0)
*(III) Flucytosine*	71(100)	0(0)	0(0)
*(IV) Amphotericin B*	71(100)	0(0)	0(0)

**Table 2:** Fluconazole and voriconazole MIC levels of *C. tropicalis* isolates determined by VITEK^®^ 2 compact system.

MIC (µg/mL)	*C. tropicalis* Isolates (*n* = 71)	Percentage (%)
**Fluconazole**		
≤1	41	57.75
2	0	0
4	4	5.63
8	13	18.31
16	1	1.41
32	10	14.08
≥64	2	2.82
**Voriconazole**		
≤0.12	43	60.56
0.25	14	19.72
0.5	9	12.68
1	3	4.23
2	2	2.82

**Table 3:** Description of biofilm production among *C. tropicalis* isolates.

Sample Type	Biofilm Non-Forming Isolates. *n* (%)	Biofilm Forming Isolates. *n* (%)		
		Weak	Moderate	Strong
Urine (*n* = 49)		4(8.16%)	22(44.9%)	23(46.94%)
Respiratory (*n* = 13)	1(7.69%)	3(23.08%)	1(7.69%)	8 61.54%)
Blood (*n* = 7)			1(14.29%)	6(85.71%)
Skin and soft tissue (*n* = 2)			1(50%)	1(50%)
Total (*n* = 71)	1(1.4%)	7(9.86%)	25(35.21%)	38(53.52%)

**Table 4:** Relationship between biofilm production and azole susceptibility.

Biofilm Formation	Azole Susceptible Isolates *n* (%)	Azole Non-Susceptible Isolates *n* (%)
No	0(0%)	1(1.4%)
Weak	7(9.86%)	0(0%)
Moderate	4(5.63%)	21(29.58%)
Strong	30(42.25%)	8(11.27%)

## Data Availability

The data presented in this study are available on request from the corresponding author.
